# Lymph vessels: the forgotten second circulation in health and disease

**DOI:** 10.1007/s00428-016-1945-6

**Published:** 2016-05-12

**Authors:** Lukasz A. Adamczyk, Kristiana Gordon, Ivana Kholová, Lorine B. Meijer-Jorna, Niklas Telinius, Patrick J. Gallagher, Allard C. van der Wal, Ulrik Baandrup

**Affiliations:** North Bristol NHS Trust, Bristol, UK; St George’s Hospital, London, UK; Pathology, Fimlab Laboratories, Tampere University Hospital, Tampere, Finland; Noordwest Ziekenhuisgroep, Alkmaar, The Netherlands; Aarhus University, Aarhus, Denmark; Bristol University, Bristol, UK; Department of Pathology, Academic Medical Centre, Amsterdam, The Netherlands; Vendsyssel Hospital, Aalborg University, Aalborg, Denmark

**Keywords:** Lymph vessels, Vascular pathology, Metastasis, Atherosclerosis, Angiogenesis, Vascular malformation, Immunohistochemistry, Genetics, Circulation, Lymphedema

## Abstract

The lymphatic circulation is still a somewhat forgotten part of the circulatory system. Despite this, novel insights in lymph angiogenesis in health and disease, application of immune markers for lymphatic growth and differentiation and also the introduction of new imaging techniques to visualize the lymphatic circulation have improved our understanding of lymphatic function in both health and disease, especially in the last decade. These achievements yield better understanding of the various manifestations of lymph oedemas and malformations, and also the patterns of lymphovascular spread of cancers. Immune markers that recognize lymphatic endothelium antigens, such as podoplanin, LYVE-1 and Prox-1, can be successfully applied in diagnostic pathology and have revealed (at least partial) lymphatic differentiation in many types of vascular lesions.

**Electronic supplementary material:**

The online version of this article (doi:10.1007/s00428-016-1945-6) contains supplementary material, which is available to authorized users.

## Introduction

A most important discovery of anatomy and physiology was Harvey’s description of the circulation of blood and a new understanding of the function of the heart in 1628. However, the full meaning of this ingenious research was first complete with the recognition of the lymphatic vasculature in the middle of the 17th century. At last, the Galenic teaching could be rejected. However, the lymphatic circulation is still a somewhat forgotten part of the circulatory system, since most research interest is devoted to the blood vessels and related diseases such as atherosclerosis and thrombosis. Nevertheless, our understanding of its function and clinical importance in both health and disease is improving, especially in the last decade. The introduction of new imaging techniques to visualize detailed anatomy and physiology of the lymphatic circulation yields a better understanding of the various manifestations of oedemas and the patterns of lymphovascular spread of cancers. New immuno markers recognize growth factors and differentiation antigens that are specific for lymphatic endothelial cells which expands the knowledge on lymphangiogenesis in chronic inflammatory diseases and also in malignant tumours. Some of these immune markers are now applied in diagnostic pathology and reveal lymphatic differentiation in an increasing number of vascular tumours or tumour-like conditions.

## Lymph vessels under normal conditions

### Macroscopic anatomy and organization

**Fig. 1:**
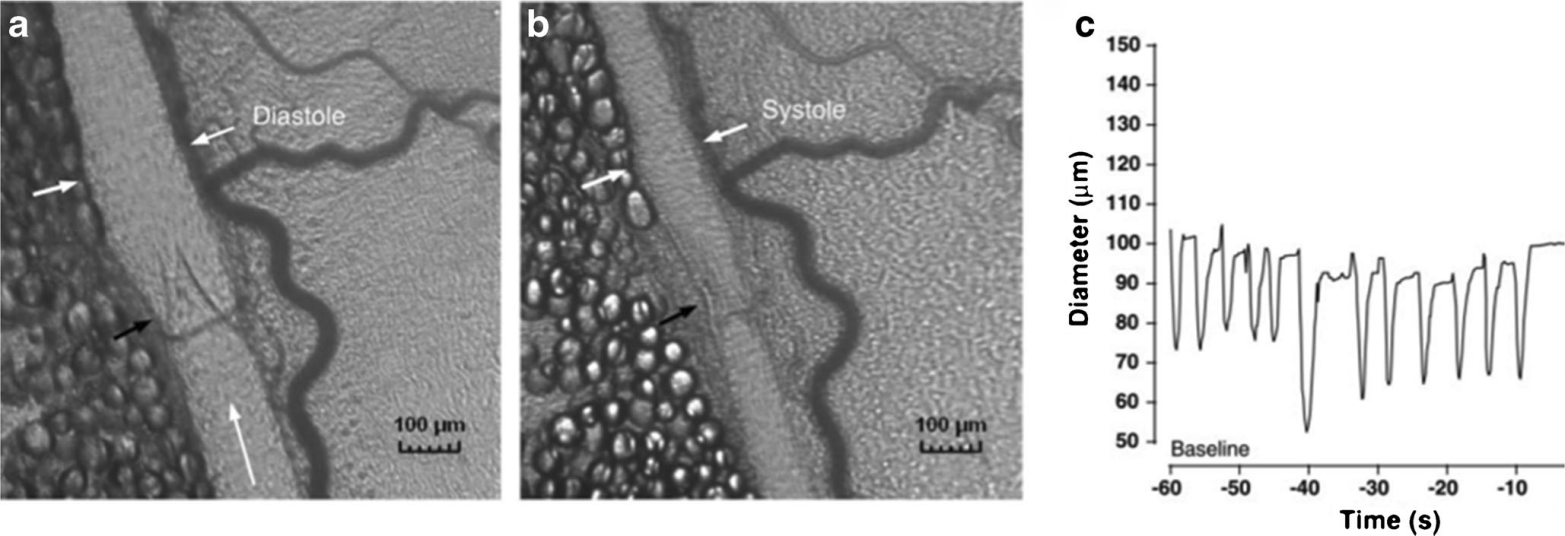
Intravital microscopy photographs showing a lymphatic vessel in diastole (**a**) and systole (**b**). The *white arrows* mark the vessel wall and the *black arrow* marks the valve. The corresponding diameter changes are depicted in (**c**). *Adapted with permission from Am J Physiol Heart Circ Physiol 2007;293:H709-18*

The lymphatic vasculature is a drainage network that begins in the interstitial spaces and ends in the great veins of the neck or thorax. The lymphatic vessels can be divided into three different types: initial lymphatics, pre-collectors and collecting lymphatics. The *initial lymphatics* are non-contracting vessels composed of a single layer of lymphatic endothelial cells attached to interstitial collagen fibres by anchoring filaments and surrounded by a discontinuous basement membrane, without pericytes [1]. Fluid entry is by absorption and depends on a pressure gradient favouring fluid movement from interstitium into the lymphatics. Further downstream, the lymph reaches the *pre-collectors*, which contain valves and sparse smooth muscle cell coverage. The pre-collectors connect the absorbing initial lymphatics to the contractile *collecting lymphatic vessels*, which have smooth muscle cells (SMCs). Unidirectional valves divide the collecting vessels into segments called *lymphangions*. These lymphangions contract spontaneously and act as pumping units and are responsible for propulsion of lymph (Fig. 1). The vessel wall is divided into three layers, tunica intima, media and adventitia, albeit less well organized than in blood vessels. Their smooth muscle cell content increases proximally. The collecting lymphatics pump the lymph further centrally, which, after passing through the regional lymph nodes, reaches the thoracic duct or the right lymphatic trunk.

The human thoracic duct is comprised of SMCs arranged in bundles with no distinct longitudinal or circumferential orientation [2]. Ultrastructurally lymphatic SMCs resemble vascular SMCs with a complete basal lamina, caveolae in regular alternation with membrane-associated dense bands, cytoplasmic dense bodies and thick (15 nm) as well as intermediate (10 nm) and thin (5 nm) filaments [2]. Interstitial Cajal-like cells have been reported in both human and sheep lymphatic vessels based on morphology and immunohistochemistry [2, 3]. Whether these cells have the role as pacemaker cells, like the interstitial cells of Cajal in the gastrointestinal tract, is not yet known.

### Physiological roles

The lymphatic system has three cardinal functions: (1) maintenance of interstitial fluid balance, (2) immune surveillance and (3) absorption of fat. All ingested fat, except the small amount of short fatty acids present in a normal diet, is taken up by the intestinal lymphatics and transported to the venous circulation. The lymphatic vessels play an important role in host defence by carrying antigens and immune cells from the tissues. It is therefore not surprising that lymphedema patients have an increased risk and severity of skin infections.

### Interstitial fluid balance

As blood passes through the capillaries, there is a continuous fluid filtration into the interstitial space. The extravasation of fluid is dictated by the Starling forces and accumulates to a total of 8 l of fluid during 24 h [4]. The only way by which the fluid can be returned to the blood circulation is via the lymphatic vasculature. Traditionally, venous reabsorption was thought to play the most important role, absorbing up to 90 % of the fluid, but there is now comprehensive evidence that venous reabsorption is non-existent in most vascular beds during steady state conditions [5]. Oedema occurs when there is a mismatch between microvascular filtration and lymphatic removal and can thus be caused by an increase in filtration, reduced lymphatic removal or both. Lymphatic failure or inadequacy is therefore present in all chronic oedemas.

### Lymph transport

Lymph transport is the result of a combination of intrinsic and extrinsic factors. Arterial pulsation, skeletal muscle contraction and respiration are examples of extrinsic factors, which cause transient tissue deformations leading to compression and expansion of the initial lymphatics. Intrinsic spontaneous contractions are generated in the lymphangions. Their contraction cycle is very similar to a cardiac ventricle. However, the heart has two pumping chambers coupled in parallel, whereas the lymphatic vessels have numerous pumping chambers coupled in series. Systolic pressure generated by the spontaneous contractions in humans can reach up to 100 mmHg, and several studies show averages of around 40–60 mmHg in arms and legs in humans, [6, 7]. The contractions are influenced by many different stimuli: humoral, neuronal, pressure, temperature and shear stress [8, 9]. Both increases in preload and afterload lead to changes in contractility [10]. Similar to arteries, there is flow-mediated NO production and myogenic constrictions as a response to increased stretch [11, 12]. Lymphatic vessels show reactivity, either inhibitory or stimulatory to a vast panel of vasoactive substances such as substance P, noradrenaline, histamine, endothelin and prostaglandins [13–15]. Despite extensive evidence of a lymphatic innervation, most consistently described as sympathetic, the exact role of the nerve supply remains unclear [16, 17].

**Table:** 

*Key points*:
• Lymphatic vessels are widely distributed throughout the body. They can be divided into absorbing, non-contracting initial lymphatics and contractile collecting lymphatics.
• Lymph flow is not a passive process, but results from spontaneous contractions generated in functional units called lymphangions

## In vivo imaging of lymphatic vessels

### Lymphoscintigraphy

**Fig. 2:**
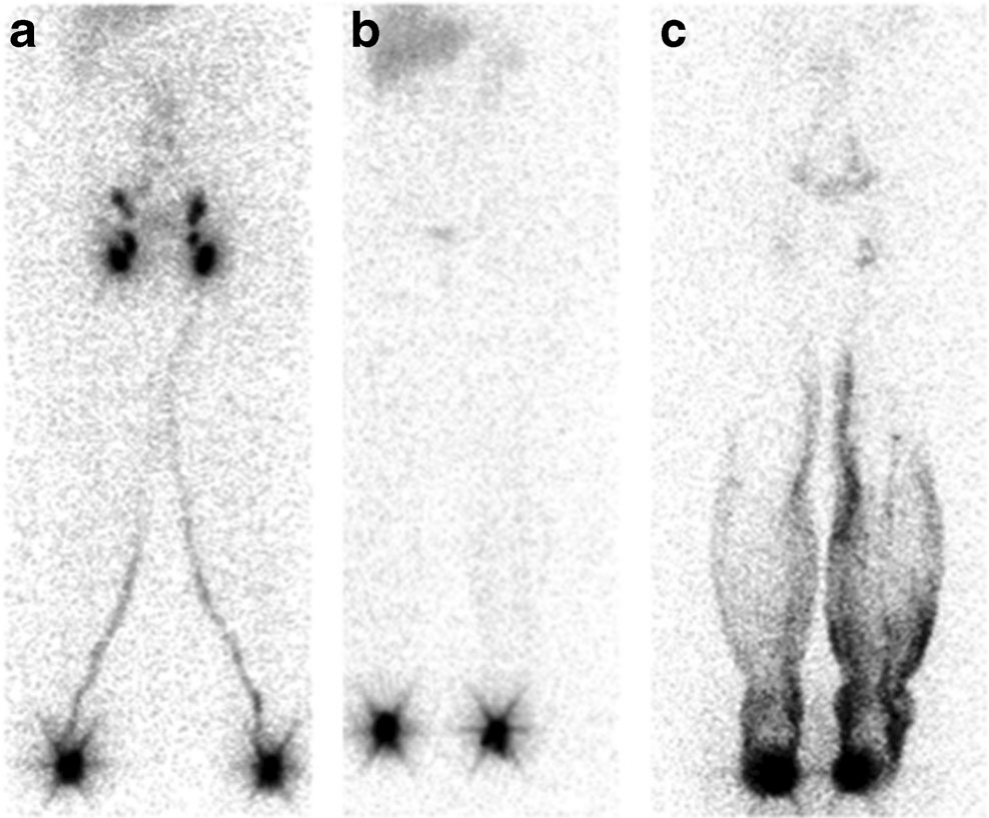
Lymphoscintigraphy of a healthy subject (**a**). Depots at the injection sites on the feet and lymphatic vessels draining the injections sites can be seen as well as the lymph nodes in the groin that the vessels lead to. Lymphatic failure in a patient with Milroy’s disease (**b**). No lymphatic vessels or uptake in the groin can be seen. Lymphoscintigraphy of a patient with lymphedema distichiasis syndrome (**c**). There is dermal reflux of lymph fluid in the lower legs. No clear vessels can be seen and there is reduced uptake in the lymph nodes in the groin

Dysfunction of the lymphatics leads to oedema. Diagnosis of the specific type of oedema is based on the history and clinical findings, eventually supported by techniques available to assess lymphatic function in a patient. Lymphoscintigraphy is the current gold standard investigation to determine whether limb swelling is the result of lymphatic dysfunction (Fig. 2) [18].

### Magnetic resonance lymphangiography

Magnetic resonance lymphangiography provides superior visualization of the lymphatic vessels, which allows better description of vessel morphology, but is inferior to scintigraphy in the detection of lymph nodes [19].

### Near infrared fluorescence imaging

Near infrared fluorescence imaging or indocyanine green lymphography is a relatively new technique used to visualize superficial lymphatic vessels [20, 21]. The technique is based on injection of a fluorescent dye that is absorbed by the lymphatic vessels. The technique is used routinely to identify suitable vessels for lymphatic-venous anastomosis surgery [22]. It is furthermore used by some to stage lymphedema [23]. The technique will probably play a key role in clinical practice and research in the future.

**Table:** 

*Key points:*
• Lymphoscintigraphy is the gold standard for investigating the lymphatic vasculature in humans. The techniques allow quantification of lymph transport and visualization of the major lymphatic vessels.
• Magnetic resonance lymphangiography and near infrared fluorescence imaging is emerging as new techniques due to superior spatial and temporal resolution.

## Primary lymphedema

Primary lymphedema is caused by a failure of the development of the lymphatic system (lymphangiogenesis) leading to either structural or functional abnormalities that impair maintenance of interstitial fluid balance. Mutations within several genes involved in the process of lymphatic development are now known to cause different primary lymphedema phenotypes. Primary lymphedema is not one disease, but the presenting feature of several distinct clinical entities.

**Fig. 3:**
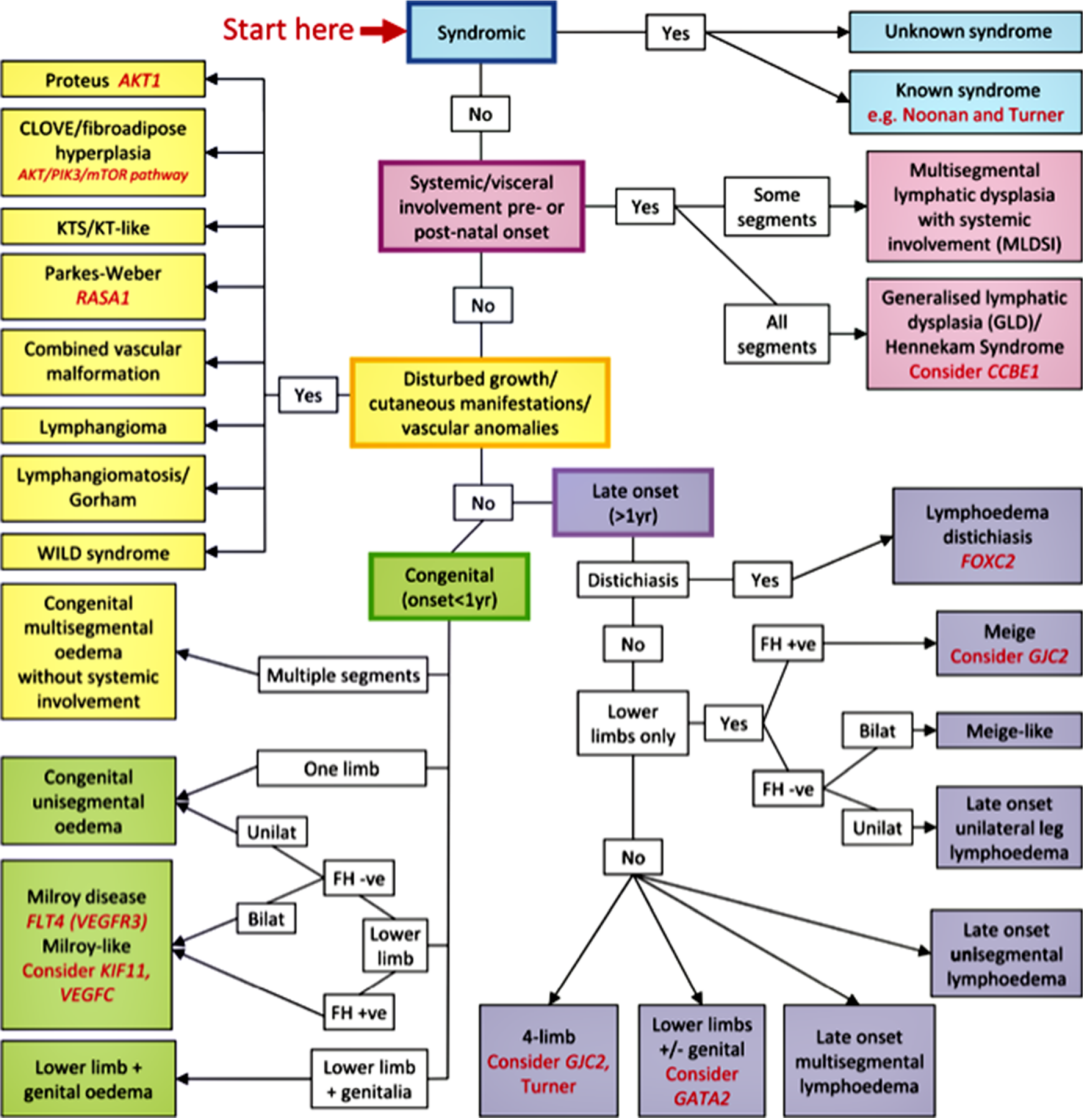
Classification pathway for primary lymphedema. The pathway provides an overview over the different primary lymphedemas, clinical features and causal genes. *Fh* = family history. Adapted with permission from *Clinical Genetics 2013;84:303–314*

Previously, primary lymphedema was classified based on age of onset: congenital, praecox (pubertal onset) and tarda (onset after 35 years). The discovery of causal genes has changed the diagnostic approach, which is now based on clinical phenotyping and genotyping in addition to age of onset of swelling (Fig. 3) [24]. Primary lymphedema can be divided into five different categories: (1) lymphedema associated with syndromic disorders (e.g. Turner or Noonan syndrome); (2) localized or generalized lymphedema with systemic/visceral lymphatic abnormalities; (3) lymphedema in association with disturbed growth and/or cutaneous/vascular anomalies (e.g. Proteus syndrome); (4) congenital lymphedema (e.g. Milroy disease); (5) late-onset primary lymphedema. A number of disease subtypes within each category have an associated causal gene. Despite recent advances in genetics, mutations in causal genes have only been detected in approximately one third of all patients with primary lymphedema [25]. For an overview over the causal genes and the resulting phenotype, see Appendix.

The identification of the genetic cause of a patient’s primary lymphedema provides a molecular diagnostic test for a number of subtypes. Nine causal genes have been discovered so far [26]. Patients and families benefit hugely from the diagnosis as it allows the clinician to confidently predict the clinical prognosis and offer screening for family members. The identification of new pathogenic genes will increase understanding of the aetiopathogenesis of lymphatic disease. In time, this may allow for the development of improved therapeutic interventions. For example, gene therapy may become a feasible treatment strategy for cases of primary lymphedema.

## Secondary lymphedema

Secondary lymphedema is the most common form of lymphedema. Globally, the parasite infection filariasis is the most common cause with over 100 million people affected. In the industrialized world, cancer-related lymphedema is the most common cause, either as a direct consequence of the cancer or the treatment.

**Table:** 

*Key points:*
• Primary lymphedema is caused by a failure of the development of the lymphatic system whereas secondary lymphedema is the result of an external stimulus, e.g. parasitic or cancer (metastasis or induced by treatment).
• Advance in genetics have so far identified nine causal genes of primary lymphedema and has helped refine the classification of primary lymphedema, which now is based on clinical genotyping and phenotyping.

## Microscopic imaging of lymphatic vessels and histopathology of lymphatics

Morphologically, it is difficult to distinguish lymphatic channels from venules or capillaries. In the era before development of lymph endothelial-specific antibodies, a variety of techniques was used to visualize lymphatic vessels, which included injection techniques with India ink or Evans Blue, hydrogen peroxide techniques [27, 28] and the enzyme histochemical detection of 5′nucleotidase [29].

### Immunohistochemical markers for lymphatic endothelium

Pan-endothelial markers such as CD31, CD34, von Willebrand factor (vWF), lectins, thrombomodulin, endoglin, and Fli-1 are expressed in all endothelial cells, including lymphatic endothelium CD31(PECAM, platelet adhesion molecule) which is considered to be the most sensitive and specific pan-endothelial marker but is also reactive with platelets and subpopulations of T lymphocytes [30, 31]. Lymphatic endothelial markers are expressed in lymphatic endothelium, but not in blood vessel endothelium. Table 1 summarizes the expression patterns of currently applied lymphendothelial-specific antibodies, which can now be applied on paraffin-embedded tissues.

**Table 1:** Expression of immunohistochemical markers for lymphatic endothelium along the vascular bed

Lymphatic markers		Podoplanin	LYVE-1	Prox-1	VEGFR-3
Lymphatic vessels	Capillary	++	++	++	++
	Collecting vessel	++	++	+	+/++
Blood vessels	Artery	-	-	-	-
	Capillary	-	-	-	+
	Vein	+	-	-	-
Hepatic sinus	+	+	-	+	
Embryonic development	+/++	+/++	++	+/++	
Postnatal development	++	++	++	++	

− :no expression; +:weak expression; ++: strong expression; +/++: variable expression

*Podoplanin* is a mucin-type transmembrane glycoprotein originally described on rat kidney podocytes [32]. In the literature, it appears also under the names AGGRUS, gp36, oncofetal antigen M2A and T1A-2 [33]. Apart from lymphatic endothelial cells, it is also expressed by other cells types including tumour cells [33]. D2-40 is the most common mouse monoclonal antibody and NZ-1 the most commonly used rat monoclonal antibody against podoplanin [33, 34].

*Prox-1* (homolog of the *Drosophila melanogaster* homeobox gene prospero) is a nuclear transcription factor, which plays a crucial role in the development of the lymphatic system. It is a master control gene introducing the expression of other lymphatic markers [35]. Immunoreactivity of Prox-1 antibody is nuclear, which is in contrast to the other lymphatic markers. Expression has been observed also in lens, heart, liver, pancreas and nervous system [36]. In colonic carcinomas, expression of Prox-1 in stem/progenitor cells is responsible for autophagy-dependent survival of metastases [37, 38].

*LYVE-1* is an integral membrane glycoprotein also known as lymphatic vessel endothelial hyaluronan receptor 1. It is an important component of extracellular matrix and a key molecule in cell migration during inflammation, wound healing and in tumorogenesis [39]. In addition to the cytoplasm of lymphatic endothelium, it is expressed in liver and spleen sinusoid endothelium and activated macrophages [40].

Vascular endothelial growth factor receptor-3 (VEGFR-3, also known as FLT4) is a membrane-anchored tyrosine kinase binding VEGF-C and VEGF-D involved in lymphangiogenesis [41]. VEGFR-3 was initially widely applied as a purely lymphatic marker, but later studies showed its reactivity with blood vessel endothelium, myoepithelial cells and cells of some non-endothelial tumours [42]. And also, other markers such as neuropilin-2, FOXC2, CCL21, D6 and aquaporin-1 appear to be not fully specific for lymphatic endothelium [43].

For studies on the (micro)vasculature in pathological conditions, we recommend the use of a panel of three endothelial antibodies, which consists of one pan-endothelial marker and two different lymphatic endothelial-specific antibodies, in order to avoid false positivity/negativity of staining results [44].

**Table:** 

*Key points*
• CD31 is the most sensitive and specific pan-endothelial marker.
• Most important specific lymphatic endothelium markers are podoplanin (D2-40), Prox-1, and LYVE-1.
• The use of the panel of one pan endothelial marker and two lymphatic markers is recommended for proper identification of lymphatic vessels.

### Lymphatic vessels: inflammation

Studies on lymph vessels in mouse models of inflammation and inflamed human tissues have shown that acute inflammatory reactions and chronic inflammatory diseases are accompanied by both the growth of new lymphatic vessels (lymphangiogenesis) and the expansion of preexisting lymphatic vessels (lymphatic hyperplasia). These mechanisms occur not only in the inflamed tissues but also in draining lymph nodes. Lymphangiogenesis, activation of lymph vessels and enhanced lymphatic drainage may have modulating effects on immune responses and the activity of the inflammatory process which is now under intense investigation [45]. This has been profoundly studied in arthritis, inflammatory bowel disease and dermatitis [46–49].

### Lymphatic vessels: transplantation

Interestingly, chronic renal transplant rejection-associated lymphatic vessel proliferation represents inflammation-associated de novo lymphangiogenesis [50]. Also, chronic cardiac allograft lymphangiogenesis is inflammation-driven and the lymph vessels are important for migration of donor and host antigen-presenting cells. There are indeed indications that suppression of lymphangiogenesis improves graft survival, for example, through blocking of the dendritic cell chemokine CCL21 in both heart and renal graft rejection [51, 52].

### Lymphatic vessels: atherosclerosis, lipid metabolism, obesity and hypertension

Lymphangiogenesis in intimal plaques and adjacent media has been reported in atherosclerotic human coronary arteries, where both the calcified and the cholesterol-rich types of plaques are involved [44]. Plaque inflammation is an important denominator in the formation of “high risk plaques” which are prone to thrombotic complications, and such an increase in lymph vessels could play a role in further recruitment of inflammatory cell into the plaque tissue. Moreover, lymphatic vessels are involved in artery wall lipoprotein metabolism and cholesterol plasma levels [53, 54]. Also, the degenerative fibrocalcified heart valves of the elderly population, which frequently display some low-grade inflammatory activity with similar phenotypic features as in atherosclerosis, appear to be prone to lymphatic growth. Aortic plaque-associated lymphatic vessel growth has also been observed in experimental mouse models of atherosclerosis [44]. Altogether, an inflammatory component in these diseases is often associated with tissue instability and related cardiovascular risk, which implies that such knowledge on lymphatic vessels can be instrumental for assessment of a potential use of antilymphangiogenic therapeutic strategies in atherosclerosis-related diseases.

## Lymphatic differentiation in tumours and tumour-like lesions

### Current classification

In practice (clinically and also pathologically) it can be difficult to determine whether a given vascular lesion is in fact a true tumour, a developmental malformation or a reactive process composed of either blood vessels, lymph vessels or both. Consistent with this, there is variability in nomenclature and sometimes misleading terminology in the literature, which may hamper appropriate evaluation and management of patients. The classification of the International Society for the Study of Vascular Anomalies (ISSVA), which stratifies the vascular anomalies into malformations and proliferative vascular lesions, has recently been updated in order to attempt a uniform state of the art and includes the main lymphatic tumours and malformations, involvement in dysmorphic syndromes and genetic backgrounds [55]. The online version is also available at www.issva.org. A previous version of this classification system has been validated recently in a large referral centre for vascular anomalies [56]. It has proven to be useful, also for clinical pathologists, albeit dependent on the adequacy of clinical information, preoperative imaging techniques and the use of immunohistochemistry for GLUT-1 (infantile haemangiomas), specific lymphatic endothelium antibodies, and the vascular smooth muscle antibody SMA-1( vascular integrity and maturation).

#### Lymphatic malformations

**Fig. 4:**
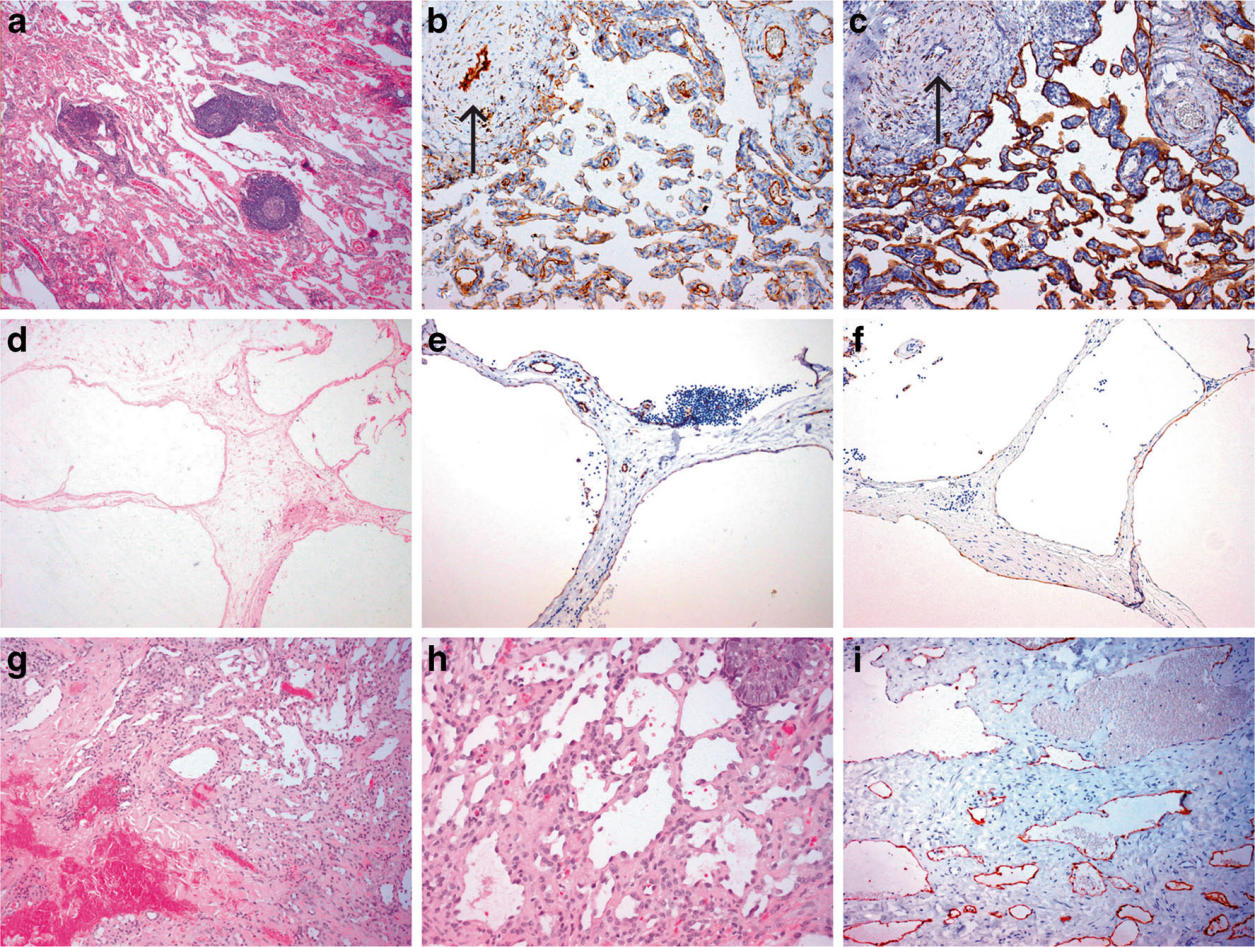
Lymphatic malformation with intralesional formation of lymph follicles (**a**–**c**); Haematoxylin and eosin stain (HE) (**a**); anti-CD31 immunostain (**b**); anti-D2-40 immunostain of endothelium (**c**); macrocystic lymphatic malformation (**d**–**e**): HE (**d**); CD31, low intensity to absent immunostaining with D2-40 antibody of endothelial cells at cystic structures (**e**) (compared with D2-140 immunostain in (**f**)); Traumatic changes and haemorrhage in LM (**g**–**h**), with hobnail-type endothelium in (**h**). D2-40 immunostaining of microvessels (**i**)

Lymphatic malformations (LMs) are sponge-like collections of abnormal and cystically dilated lymph vessels filled with lymph fluid. In the past, most lymphatic lesions were designated as lymphangiomas’, but in the current classification, they now are labelled as ‘lymphatic malformations’ because of the usually congenital nature, slowly progressive growth over time and composition of malformed dilated lymphatic vessels [55, 57]. Based on the size of vessel lumen, LMs are classified as microcystic (previously termed lymphangioma circumscriptum), macrocystic (previously termed cystic hygromas) or mixed subtypes [58]. They can be located superficially or deep and occur (multi) focal or diffuse. Sudden enlargement may occur during infection or spontaneous bleeding within the cysts. Many lesions are congenital, although not always visible at birth, but small lesions may develop also in response to trauma or infection. Lymphangiogenic growth factors, such as VEGF-C and VEGFR-3, are upregulated in LMs, but the precise role of these factors in their development is not clear [59]. In histopathology, an LM reveals a convolute of dilated lymph vessels with incomplete medial layers of one to multilayered smooth muscle cells and can be associated with nodular lymphoid tissue (Fig. 4a–c). Endothelial cell lining is flat but obtains a hobnail-like appearance, in cases with trauma, haemorrhage or chronic inflammation (Fig. 1). Endothelium stains positively with several lymphatic specific antibodies [60], but staining intensity can be markedly reduced in the flat endothelium of cystically dilated lymph channels (Fig. 4d–k). Traumatic changes in lymphatic malformations frequently cause haemorrhage and thrombosis, especially in combined lymphaticovenous malformations. Therefore, presence of erythrocytes in vessel lumina does not rule out a diagnosis of a lymphatic lesion. Additional hamartomatous tissue components such as fat, nerve bundles or of reactive microvascular proliferations, which are frequently found in congenital malformations of blood vessels, are not a feature of pure lymphatic malformations [61, 62].

### Vascular tumours with lymphatic differentiation

**Fig. 5:**
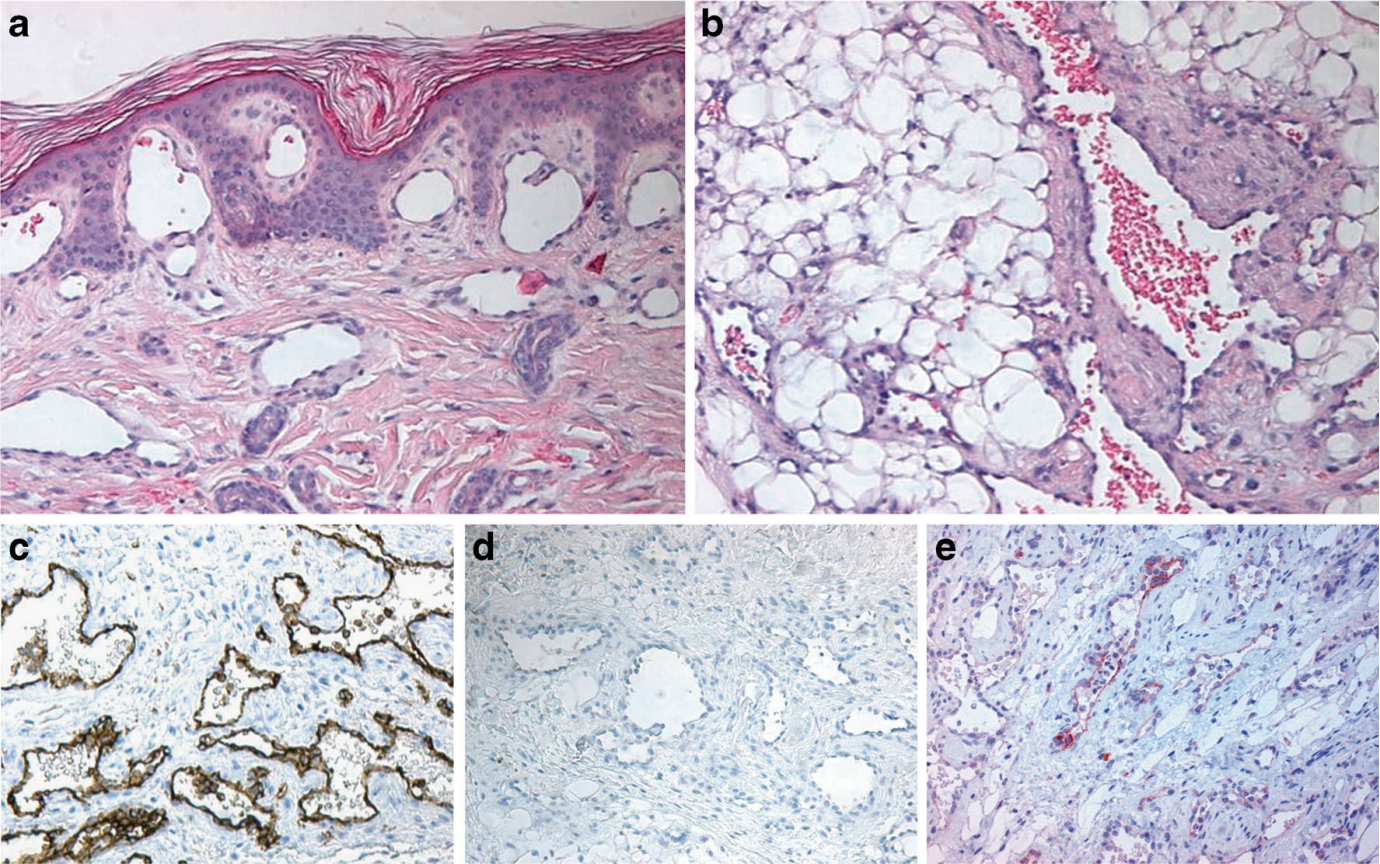
Multiple lymphangioendotheliomatosis. Skin surface with subepidermal dilated thin-walled vessels (HE) (**a**); deep-seated atypical vessels in subcutis, partially filled with erythrocytes (HE) (**b**); CD31 immunostain of atypical microvessels (**c**); absent D2-40 immunostaining (**d**); focal LYVE-1 immunostaining of microvessels (**e**)

The use of monoclonal antibodies specific for lymphatic endothelium has revealed lymphatic endothelial differentiation, at least to some extent, in a still increasing list of vascular tumours that were initially considered to be composed of blood vessels only (Table 2). However, in many of these lesions, the patterns of immunostaining vary when different lymphatic antibodies are applied (see for example Fig. 5). Therefore, it is recommended to use two different lymphatic antibodies; salient examples of such ‘newly’ described lymphatic lesions are as follows:

**Table 2:** Tumours and tumour-like lesions with at least partial lymphatic differentiation

Name	Biological behavior according to ISSVA^a^ 2015 [55]
Primary lymphoedema/lymphatic malformation	Congenital malformation. Isolated or in combination with other vascular or nonvascular malformations or genetic syndromes
Tufted angioma	Benign tumor of skin. Partial lymphatic differentiation^b^
Spindle cell angioma	Provisionally unclassified. Probably vascular malformation with partial lymphatic differentiation
Verrucous angioma	Provisionally unclassified; probably congenital malformation with partial lymphatic
Angiokeratoma	Provisionally unclassified vascular anomaly. Partial lymphatic differentiation
dMLT/CAT^c^	Provisionally unclassified lesion.
Kaposiform hemangioendothelioma	Locally aggressive tumor. Extends into deep tissues; resembles tufted angioma
PILA^d^	Borderline malignant tumour
Kaposi sarcoma	Borderline malignant tumour
Lymphangiosarcoma	Malignant. Variable expression patterns with lymphatic markers
Kaposiform lymphangiomatosis	Provisionally unclassified vascular lesion

^a^International Society for the Study of Vascular Anomalies

^b^Partially lymphatic indicates: composed of blood vessels and lymph vessels

^c^Diffuse multiple lymphangioendotheliomatosis

^d^Papillary intralymphatic angioendothelioma (Dabska tumour)

1. Spindle cell haemangioma. First reported in 1986 as low-grade malignant and termed spindle cell hemangioendotheliomas [63]. It is now considered as a benign type of lesion with clinical and biological features more reminiscent of congenital vascular malformations. A recent study of 12 cases of spindle cell haemangiomas showed a positive endothelial lining with coexpression of pan-endothelial and lymphatic antibodies, suggesting its lymphatic origin [64].
2. Multiple lymphangioendotheliomatosis. An extremely rare disease in infancy associated with thrombocytopenia [65]. Patients present with hundreds of congenital vascular lesions in the skin, which have the characteristics of both blood vascular and lymphatic vessels (Fig. 5). Similar lesions involve the gastrointestinal (GI) tract causing severe GI tract bleeding.
3. Kaposiform hemangioendothelioma (KHE) of childhood. This is a vascular tumour with a locally aggressive behaviour and often complicated by severe coagulopathies (in the most serious form, the Kasabach-Merritt syndrome) [66]. However, these coagulation abnormalities are not unique for KHE but have been noticed also in other vascular tumours and malformations [56]. Spindle cells of this tumour coexpress blood and lymphovascular markers [67].
4. Kaposi sarcomas (KS). Also coexpress blood vascular and lymphatic markers in all the histopathological variants. These markers are useful in the recognition of KS, since there is a variation in morphology of the lesions varying from scant multicentric, small dilated microvessels to solid spindle cell proliferations; others present as polypoid tumour masses mimicking pyogenic granuloma or even closely resembling haemangiomas. Recent studies have shown that the KS herpes virus can infect endothelial cells of blood vessels and reprogram the neoplastic HHV8-infected endothelial cells to lymphatic endothelium. The tumour cells show induction of lymphatic lineage-specific genes, including Prox1 and downregulation of blood vascular genes [68, 69].
5. Angiosarcomas. Application of lymph endothelial specific antibodies has revealed at least partial lymphatic differentiation in a large subset of lesions that were traditionally considered to be true hemangiosarcomas (both the solitary and the radiation induced types of tumours). In a recent study of 49 angiosarcomas by Mankey et al. [70], more than half of the number of tumours showed immunopositivity with at least two out of three applied lymphatic antibodies (D2-40, Prox 1, LYVE-1). Especially, tumours with intratumoral aggregates of lymphoid tissue and prominent hobnail appearance of endothelium showed broadest immunoreactivity with these markers. This implies that at least histopathologically, they should be defined as lymphangiosarcomas.

**Table:** 

*Key points*
• Many lesions that were previously designated as ‘lymphangiomas’ are in fact lymphatic malformations, being mostly congenital in nature
• Specific lymphatic endothelium antibodies identify (partial) lymphatic differentiation in a still increasing number of vascular tumours
• Occurrence of blood-filled spaces, haemorrhages or hobnail appearance of endothelium does not rule out the diagnosis of a lymphatic lesion

## Lymphovascular invasion: patterns and diagnosis

The importance of vascular invasion in tumour biology was established over 100 years ago. In his classic textbook, Willis summarized the early work and described the patterns of tumour spread in autopsies on patients with a variety of disseminated cancers [71]. From the numerous recent studies on lymphatic and blood vessel invasion (LBVI) in surgically resected tumours, we have summarized reports which emphasize the importance of accurate detection of vascular invasion (Table 3). Many of these reports indicate no clear distinction between lymphatic and blood vessel invasion or do not focus on lymphatic vessels in depth. In everyday practice, the detection of lymphatic invasion (LI) alone is especially important in breast carcinoma and malignant melanoma in particular because it may influence on patient management or the choice of adjuvant chemotherapy [78, 80, 81].

**Table 3:** Representative studies that emphasise the importance of lymphovascular invasion in surgical resections listed chronologically

Authors, setting and date of study (reference number)	Number and type of biopsy	Principle findings	Comments
Alexander-Sefre et al. London, UK 2003 [72]	108 patients with stage 1 endometrial adenocarcinoma	Substantial increase in the detection of vascular invasion with pan-cytokeratin and CD31 immunohistochemistry (from 21 to 58 cases). No distinction between blood and lymphatic vessels	Vascular invasion has been underestimated in early endometrial adenocarcinoma
Vass et al. Glasgow, UK 2004 [73]	75 Colonic adenocarcinomas	Elastin staining increased detection of venous invasion from 18 to 32 cases for extramural invasion and from 8 to 30 cases for intramural invasion	Elastic staining should be part of standard protocols
Pawlik et al. China, France, Japan, USA 2006 [74]	Multinational registry of 1073 resections for hepatocellular carcinoma	41 % of tumours >5 cm had LBVI in comparison with 27 % <5 cm. Multicentricity, histological grade and high AFP levels also associated with LBVI	No comments on histological methods for identifying LBVI
Chen et al. South Australia 2010 [75]	110 Whipple’s resections for pancreatic carcinoma between 1998 and 2008	5 year survival 77 % in patients negative for both LBVI and perineurial invasion but only 15 % in patients positive for both	Poor differentiation, size >3 cm and nodal involvement also poor prognostic features
Storr et al. Nottingham, UK 2012 [76]	202 cutaneous melanomas	Lymphatic invasion more common than venous invasion (27 vs 4 %). Immunohistochemistry with CD34 and D2-40 increases detection rate of blood vessel and lymphatic invasion.	Lymphatic invasion is associated with adverse factors but lymphatic characteristics do not predict outcome
Kirsch et al. Canada 2013 [77]	Sections of 40 colorectal carcinomas circulated to specialist and non-specialist GI pathologists	GI pathologists detected venous invasion more frequently than non-GI with both H&E and Movat’s stain. Detection of venous invasion was >2 fold higher with Movat’s stain (46 vs 20 %)	Venous invasion is under-detected with H&E, even by specialist pathologists
Gujam et al. Glasgow, UK 2014 [78]	Review of 59 reports of 62514 patients with breast carcinoma	19/21 studies demonstrated lymphatic vessel invasion predicted poorer prognosis. Improvement of lymphatic detection using immunohistochemistry	Guidelines for the use of immunohistochemistry in mammary carcinoma should be followed
Castonguay et al. Canada 2014 [79]	103 oesophageal adenocarcinoma resections	Venous invasion detected in 8 cases with H&E but in an additional 66 cases with Movat’s pentachrome	Venous invasion, stage, size and grade prognostically significant on univariate analysis

Expressed in haemopoetic and vascular associated tissues, *D2-40*—an antibody directed against a glycoprotein selectively expressed on lymphatic endothelium; Movat’s pentachrome—a modification of the trichrome method incorporating specific staining of elastic tissue, widely used by cardiovascular pathologists especially in North America

*CD 31* platelet endothelial cell adhesion molecule, *CD 34* a cell surface glycoprotein of uncertain function, *GI* gastrointestinal, *BLVI* lymphovascular invasion, *H&E* haematoxylin and eosin

### Detection of lymphovascular invasion in surgical resections

1. Breast
  LBVI is underdetected in breast carcinoma, probably in as many as a fifth of cases. Blood vessel and lymphatic endothelial markers are helpful in detection of LBVI both within and at the edge of tumours. Invasion in vessels at the advancing tumour edge is especially important [81]. In a recent review of more than 30 reports, Gujam et al. found that blood or lymphatic vessel invasion was detected in 24 % of cases on H&E rising to 35 % with immunohistochemistry [78]. Although the exact prognostic significance of vascular invasion is uncertain, its presence or absence may influence the type of chemotherapy that is used. The review emphasizes that the detection of LI is especially important in operable breast carcinomas without lymph node spread. However, some studies indicate LBVI in node-positive breast carcinoma may be prognostically valuable.
2. Oesophageal and gastric carcinomas
  The incidence of oesophageal and gastric carcinomas is increasing in many countries and the 5-year survival is persistently low. In some studies of oesophageal carcinoma, venous invasion was found to reduce both disease-free survival and cancer-specific survival [82]. Recognition of any type of vascular invasion in oesophageal carcinoma is now a requirement and distinction between lymphatic and venous may be relevant [83].
  In earlier studies, gastric cancer LBVI was independently related to survival and cancer recurrence [84] regardless of tumour stage or size [85]. LI is a common feature in gastric cancer but despite early suggestions of lymphatic invasion alone playing an independent prognostic role [86], this hypothesis requires further confirmation.
3. Colorectal cancer
  The latest colorectal cancer dataset of the Royal College of Pathologists of Great Britain and Ireland incorporates assessment of intramural and extramural vascular invasion and the distinction of lymphatic and venous involvement in colorectal carcinoma [87]. Lymphatic invasion was suggested as an adverse factor for survival more than 20 years ago [88] but the current prognostic emphasis is put on extramural venous invasion. It is especially important to identify LBVI in Dukes B cancers where its status may guide decisions on adjuvant therapy [89].
  Specialists in GI pathology report LBVI more frequently than do general pathologists (30 vs 9 %) but even they increase their rate of detection when elastin stains are used (Table 1) [77]. A recent study by Kojima et al. showed marked improvement of detection of LBVI including LI after agreement of set microscopic criteria after a series of questionnaires and consensus meetings between pathologists (Delphi method). Two features reaching the highest kappa value in the study were either a rim of elastin or D2-40 marking cells covering more than 50 % circumference of a tumour cluster [90].
4. Malignant melanoma
  There is currently well-documented preferential LI in cutaneous malignant melanoma. In the recent work, Rose et al. examined 246 cases of malignant melanoma and showed D2-40 marker staining increased detection rate of LI sixfold compared with routine H&E. After statistical evaluation, LI remained an independent prognostic factor for adverse patient prognosis [80]. In a UK multicentre study, Storr et al. showed over a quarter of examined cases were positive for LI compared with only 4 % of invaded blood vessels. LI correlated with mitotic rate, ulceration and Breslow thickness but statistically failed to reach independent prognostic significance [76].

### Techniques for detection of lymphatic and venous invasion

The studies discussed above and summarized in Table 3 emphasize the importance of LBVI and stress that it is often underdiagnosed. Three different approaches can be used to increase the accurate detection of invasion.

**Fig. 6:**
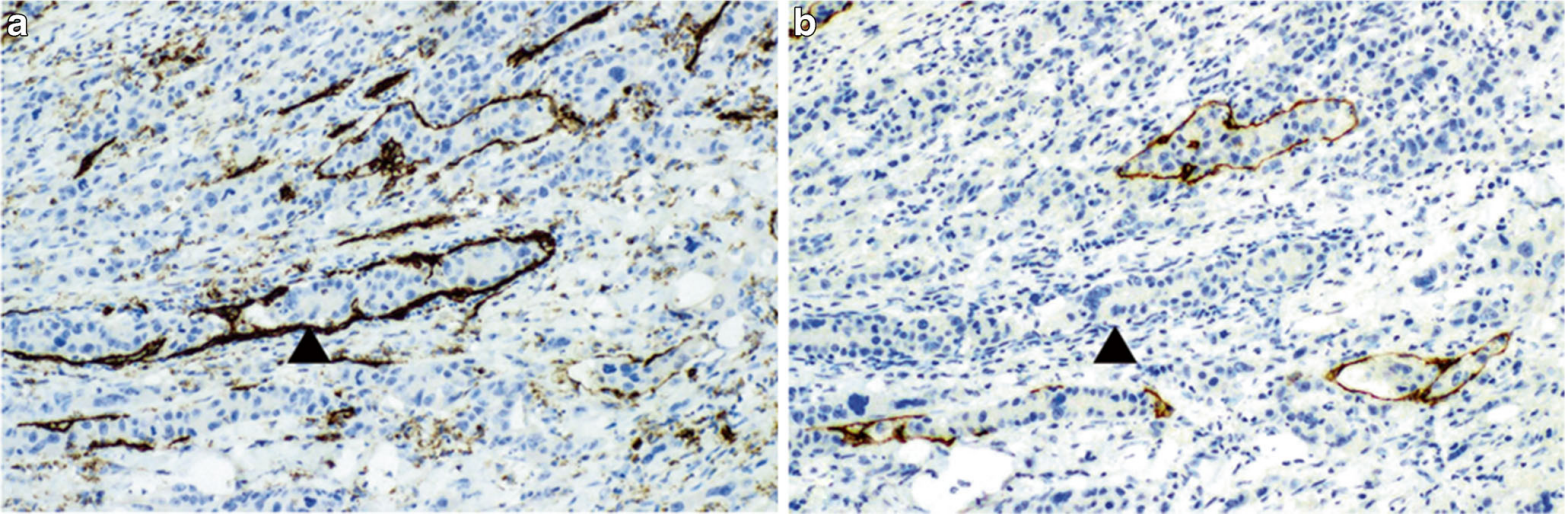
Detail of tumour (melanoma) with lymphovascular invasion; CD31 immunostain showing immunoreactivity of endothelium in all microvessels (**a**); D2-40 immunostain showing immunoreactivity of endothelium with intravascular tumour deposit (**b**)

- Appropriate sampling of tumours and adjacent tissues
  Few studies have specifically addressed dissection techniques for the enhanced detection of LBVI. The convention that malignant tumours should be sampled in three blocks is outdated and we advocate a minimum of five. Sternberg and colleagues dissected blocks perpendicular and tangential to tumours, blocks taken across the mesentery and around major blood vessels [91]. The authors found that LBVI was most often detected in perpendicular blocks but in some cases, it was only identified in the tangential blocks. Further studies of dissection techniques and the number of blocks that should be sampled would be valuable.
- “What to look for”—recognition of the particular histological features of vascular invasion
  The multicentre Canadian study of Kirsch et al. described two important histological signs that suggest LBVI in colorectal carcinoma, although no distinction was made between lymphatic and blood vessels. Veins and arteries are closely related to each other but this is especially true in the bowel wall. The so-called protruding tongue sign is a rounded protrusion of carcinoma extending towards the extramural space at the tumour edge. This suggests venous invasion, especially if the deposit is close to a small artery. In the ‘lone artery’ or ‘orphan arteriole’ sign, an often elongated tumour deposit is seen next to a normal extramural artery. This, in fact, is an obliterated vein twinned with the artery [77]. Whether this also applies to lymph vessels is not known.
- Routine use of special stains, especially elastic tissue stains and vascular immunohistochemistry.
  Small- and medium-sized lymphatics contain a network of elastic tissue although less in abundance than in veins. In contrast to arteries, this is not arranged as distinct internal and external lamellae but as an irregular network. Small lymphatic invasion can be identified with immunomarkers but larger vessel invasion might be indistinguishable from venous as the endothelium is obliterated by most carcinomas. Elastin can form tumour within nests of malignant cells and must not be mistaken as evidence of vascular invasion. In this context, Kojima et al. suggested that venous invasion should only be diagnosed when a tumour deposit has 50 % of its circumference surrounded by elastin or D2-40 [90]. Remnants of vascular media of larger veins or lymph vessels can sometimes be visualized adjacent to tumour deposits with the use of smooth muscle actin immunostaining. Figure 6 displays intratumoral invasion of small vessels by immunostaining for CD31 and D2-40.

Representative studies that emphasize use of LVI in surgical resections are listed in Table 3. Multiple studies of gastro-oesophageal and colonic carcinomas have shown that intramural and extramural LBVI is easily missed. Routine and inexpensive elastic tissue stains increase the diagnostic accuracy of both specialist and non-specialist pathologists. We suggest that they should be used routinely. In most reports, no clear distinction is made between lymphatic and blood vessel invasion. However, for certain tumours, especially melanomas, this appears to be relevant as shown in recent studies where immunohistochemistry with specific endothelial antibodies was applied to discriminate between lymphatic and blood microvasculature. These techniques at least increase the detection of venous and lymphatic invasion in mammary carcinoma and in melanoma. They should be used in selected cases, especially node-negative operable tumours.

**Table:** 

*Key points*
• LI detection in breast carcinomas is especially important in patients with operable breast cancer without lymph node involvement.
• Defined criteria of vascular invasion need to be used alongside special stains to detect vascular invasion in colorectal carcinoma.
• Vascular antibodies, particularly the lymphatic marker D2-40, improve detection of LI in malignant melanoma.

## Electronic supplementary material

Below is the link to the electronic supplementary material.

ESM 1 (DOC 26 kb)
