# Supplementary material for: Lymph vessels: the forgotten second circulation in health and disease
Source: Virchows Arch. 2016 May 12;469:3–17. doi: 10.1007/s00428-016-1945-6 (PMC4923112; doi:10.1007/s00428-016-1945-6)
Supplement: Supplementary file 1 — (DOC 26 kb) [file 428_2016_1945_MOESM1_ESM.doc]

**APPENDIX**

**Lymphatic Pathology and Molecular Genetics**

Causal mutations can be divided into two groups: genes known to be involved in regulation of lymphangiogenesis; and those not known to be involved in lymphangiogenesis. Nine causal genes for primary lymphedema have been identified so far [92]. Mutations in genes involved in lymphangiogenesis include vascular endothelial growth factor receptor type 3 (VEGFR3), vascular endothelial growth factor C (VEGFC), collagen and calcium binding EGF domains 1 protein (CCBE1), SOX18 and forkhead box protein C2 (FOXC2). Vascular endothelial growth factor receptor type 3 plays an important role in the lymphangiogenesis pathway [93]. Mutations in the VEGFR3 gene cause Milroy Disease and this was the first causal gene of primary lymphedema to be identified [94]. Milroy Disease typically presents with congenital bilateral lower limb oedema caused by hypoplasia of initial lymphatics and consequent failure to absorb interstitial fluid. Mutations in VEGFC, the ligand to VEGFR3, cause a pattern of primary lymphoedema that is similar to Milroy Disease [95]. Lymphoedema distichiasis syndrome was the second subtype of primary lymphoedema where an underlying causal gene was identified. The syndrome is characterized by pubertal onset of bilateral lower limb lymphoedema, and aberrant eyelashes arising from the meiboman glands. The syndrome is caused by a mutation in the FOXC2 gene, which is responsible for the development of valves in both lymphatic vessels and veins [96,97]. Lymphoscintigraphy demonstrates dermal reflux of lymph fluid in the lower legs due to the valve failure. The oedema is augmented by hyperfiltration of interstitial fluid due to venous insufficiency.

Kinesin family member 11 (KIF11), GATA2 and AKT1 pathway gene mutations also cause primary lymphedema but their role in lymphangiogenesis remains unclear. Microcephaly with or without chorioretinopathy, lymphedema, and intellectual disability (MCLID) occurs as a result of mutations in the KIF11 gene and presents with lymphedema identical to Milroy Disease [98]. Similarly, GATA2 was identified by Ostergaard and colleagues in 2011 to cause Emberger syndrome, i.e. primary lymphedema and myelodysplasia [99]. The mechanism of action upon lymphangiogenesis remains unclear, as GATA2 is an established regulator of haematopoiesis.

References

92. Mortimer PS, Rockson SG (2014) New developments in clinical aspects of lymphatic disease. J Clin Invest 124:915–921

93. Kaipainen A, Korhonen J, Mustonen T, et al (1995) Expression of the fms-like tyrosine kinase 4 gene becomes restricted to lymphatic endothelium during development. Proc Natl Acad Sci USA 92:3566–3570

94. Ferrell RE, Levinson KL, Esman JH, et al (1998) Hereditary lymphedema: evidence for linkage and genetic heterogeneity. Hum Mol Genet 7:2073–2078.

95. Gordon K, Schulte D, Brice G, et al (2013) Mutation in vascular endothelial growth factor-C, a ligand for vascular endothelial growth factor receptor-3, is associated with autosomal dominant milroy-like primary lymphedema. Circ Res 112:956–960

96. Fang J, Dagenais SL, Erickson RP, et al (2000) Mutations in FOXC2 (MFH-1), a forkhead family transcription factor, are responsible for the hereditary lymphedema-distichiasis syndrome. Am J Hum Genet 67:1382–1388

97. Brice G, Mansour S, Bell R, et al (2002) Analysis of the phenotypic abnormalities in lymphoedema-distichiasis syndrome in 74 patients with FOXC2 mutations or linkage to 16q24. J Med Genet 39:478–483

98. Ostergaard P, Simpson MA, Mendola A, et al (2012) Mutations in KIF11 cause autosomal-dominant microcephaly variably associated with congenital lymphedema and chorioretinopathy. Am J Hum Genet 90:356–362

99. Ostergaard P, Simpson MA, Connell FC, et al (2011) Mutations in GATA2 cause primary lymphedema associated with a predisposition to acute myeloid leukemia (Emberger syndrome). Nat Genet 43:929–931
